# Pattern and outcome of children admitted for burns in Benin City, mid-western Nigeria

**DOI:** 10.4103/0970-0358.59279

**Published:** 2009

**Authors:** O. O. Oludiran, P. F. A. Umebese

**Affiliations:** Department of Orthopaedics and Traumatology, College of Medical Sciences, University of Benin, Benin City, Nigeria

**Keywords:** Burns, kerosene explosions, scalds

## Abstract

Children are a vulnerable to burns, an injury, which is often preventable. A study of the profile of cases of children admitted for burns will provide background information to suggest locally doable preventive strategies as well as supply basic information for future reference. We studied the records of 62 children aged 0-16 years, admitted for burns, at the University of Benin Teaching Hospital, Benin City, between January 2002 and December 2006. There were 34 male and 28 female children. Children under three years constituted 56.5%. Whereas the leading cause of burns in all the children was flame burns from kerosene explosions (52%), scalds were responsible for 68.6% of cases in those under three. The extent of burn injury ranged from 6 to 50% and most of them presented late. 64.6% were discharged within three weeks. Wound sepsis and post burn contractures were the most frequently encountered complications (19.4% and 9.7% respectively). There were two deaths (3.2%) related to sepsis. Particular attention to burn safety precautions in children (especially, in the >3 years age group), safer storage and dispensing of combustible chemicals particularly petroleum products is advocated. Fire safety awareness, correct first aid measures and early presentation in the hospital will reduce morbidity and mortality. Early physiotherapy and splinting strategies will reduce contractures. There is the need locally for the establishment of specialized burn centres both to treat these children and to stimulate interest in burn management.

## INTRODUCTION

Burn injury remains a major cause of morbidity and mortality in the developing countries. Inadequate public education, injury prevention and control measures coupled with lack of coordinated rescue effort in the face of poorly funded health facility add to compound the lifelong deformity and disability, that are the aftermath of burn injury. Thus, it is not uncommon to encounter gross post burn deformity in plastic surgical clinics in the developing world. These injuries and the ensuing deformities cost much less to prevent than to correct. An understanding of the epidemiology of burns in children is essential for a focused burn prevention strategy applicable to our local environment; as well as in similar situations elsewhere. Unfortunately, relevant and accurate data regarding these injuries is sketchy and incomplete, particularly in the developing countries. Where present, such data are from well established burn centres in developed cities remote from the realities of rural and suburban life.

Children represent a subset of the population at risk of burns particularly in the domestic setting where effective and actionable preventive measures can be designed and monitored. Unfortunately, they still form a major part of the burn casualty. They accounted for 38 and 39% of all burns cases seen at Ibadan and Cairo respectively.[1] Similarly, Chien and colleagues, in Taiwan, described two peak incidences at 0-5 yrs and 35- 44yrs in their series.[2] There were disproportionately more children in Blantyre, Malawi than adults.[3] In most of the series scalds were the most common cause of injury which is consistent with what is most widely believed.[2,3]

At the University of Benin Teaching Hospital, burn care had been provided by paediatric, orthopaedic and trauma surgeons until recently when the plastic surgery unit was established. There is no local study which has defined the injury burden from burns in children. This study was carried out to determine the causes and severity of burn injury among children admitted into the burn services of the University of Benin Teaching Hospital and to compare same with similar reports from other centres.

## MATERIAL AND METHODS

We reviewed the ward admission records, case notes and medical charts of all children aged 0-16 years admitted into the surgical services of the University of Benin Teaching Hospital from January 2002 to December 2006 for burns. These were supplemented with information obtained from the nurses' change and report books. Information obtained include patients basic demographic data, cause and circumstance of burns, time lapse from injury to presentation into our health institution, percentage total surface area burnt, injury distribution by regions, duration of hospitalization, outcome of management and outpatient follow up.

Until recently, all burn cases were managed in a general care setting by surgical residents under the care of an orthopaedic and trauma consultant surgeon. The same admission facility serves other children admitted for surgery: paediatric, maxillofacial, orthopaedic and ENT surgery as well as paediatric urology.

All thermal burns were admitted through the Accident and Emergency section of the hospital where initial airway management (except intubation), fluid resuscitation by the Parkland formula and wound dressings with dermazin after cleansing with saline were carried out. Analgesics and antitetanus prophylaxis were administered. Antibiotics were often prescribed. Facial wounds were treated with antibiotic ointment and left exposed. Operative interventions included early burn wound excision for full thickness and septic burn wounds. We selectively covered joint areas, the face and the hands with split thickness autografts when the burn is obviously full thickness or it showed no signs of epithelialization two weeks post burn.

The physiotherapists were involved throughout the course of management for chest physiotherapy and both active and passive joint ranging.

## RESULTS

**Figure 1:**
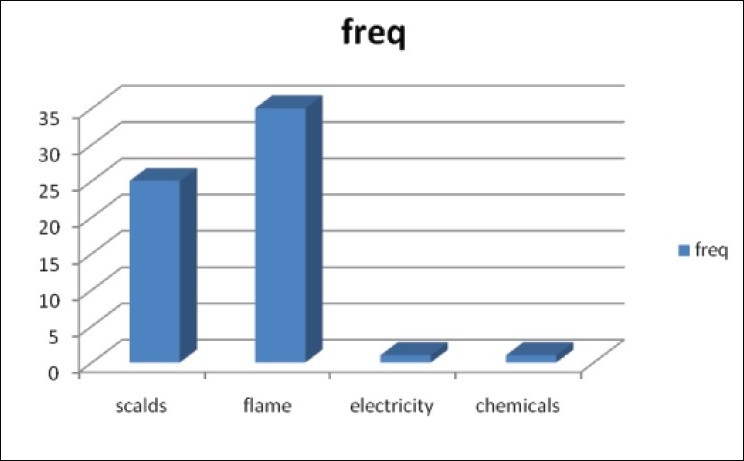
Cause of Burns in All Children

**Figure 2:**
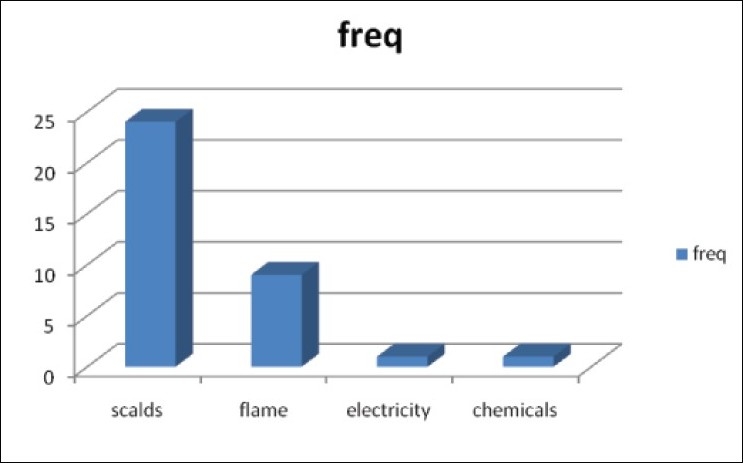
Cause of Burns in Children 3 Years and Below

**Figure 3:**
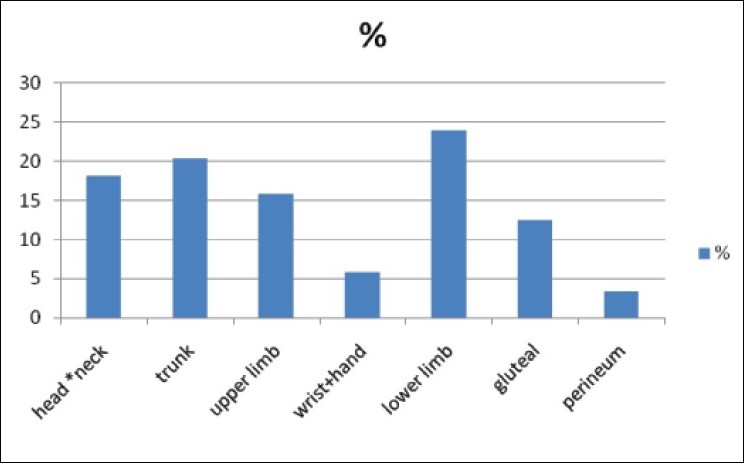
Regional Distribution of Burn Injury

There were 62 children admitted during the study period for burns. Of these, 34 were males and 28 females. They were aged three weeks to sixteen years (mean = 5.1+/− 4.3). Children under three years were more affected than those above 3 (35: 27) [Table 1]. Whereas flame burns from kerosene lamp explosion was the most common mode of injury (32/ 62, 52%), children under three suffered more from scalds (24/35; 68.6 %) [Figures 1 and 2]. There was only one case of electrical burn and another of chemical burns from acetic acid. The time lapse from injury to presentation ranged from 10 minutes to three weeks. Only seven patients presented in the hospital within the first three hours post burn. Fifty five children had their burn surface area properly assessed and documented [Figure 3]. The percentage burnt surface area was from 6- 50% [Table 2]. The burn depths were not adequately documented for proper evaluation. Duration of hospitalization in our series ranged from three to forty three days. Forty patients (64.5%) were discharged within three weeks of admission. Complications were recorded in 20 patients: wound sepsis in 12 (19.4%), 6 developed contractures (9.7%) and one patient each had acute renal failure and burn syndactyly, respectively.

**Table 1:** Age Distribution

*Age (Yr)*	*Frequency*
1	6
3	29
4- 6	11
7- 10	9
10	7
Total	62

**Table 2:** Extent of Burns (%TBSA) (No = 55)

*% TBSA*	*Frequency*
< 10	12
11- 20	23
21-30	10
31- 40	5
41- 50	5

There were two deaths (3.2%) and two other children were discharged by their parents against medical advice. The two deaths were caused by septicaemia.

## DISCUSSION

This study was carried out to describe the epidemiological and clinical profile of children with burn injury severe enough to necessitate in hospital care in our institution over a five year period. Sixty two children were admitted showing a hospital annual admission incidence of 12 cases a year with a slight male preponderance. These are fewer compared to the number of children admitted in other parts of our country in the series reported by Gali *et al*[4] and Mungadi[5] from Northern parts of Nigeria; we however, note a similarity in the sex distribution of the patients. It is difficult to conclude that our children are less vulnerable. One feature, which may not be unique to our environment, is that most patients do not seek medical care unless they have evidently life threatening conditions or severe intractable pains. Medical service has been loosely regulated with poor service coordination and disease reporting. It is probable that a large number of patients are treated outside our centre. The fact that many of the patients are seen with complications in the post acute period lends credence to this. Indeed, since the commencement of the plastic surgery unit, more cases are being attended to every month and this is expected to increase when the burns unit become fully operational.

Children under three constituted 56.5% of the cases admitted during the study period. We stratified the respondents into three year age range with the understanding of the local enrolment pattern of children: preschool care and elementary schools. Most children are in the day care play centres before three, attend kindergarten school till five or six years before they enrol in the primary school. Thus, under- three children are directly under the care of the parents or some foster home. This age is the period when children begin independent mobility, manifest exploratory and hand to mouth behaviour. The pattern in this age group agrees with local and foreign studies.[6–9] In this age group, most burns were due to scalds from hot water, soup or rice water. Indeed, only one child older than three had burns from scalds in our series. Leaving children unattended to during this vulnerable period exposes them to this hazard.

Flame burns are the most common cause of injury, accounting for 52.0% in our series in all children. This is due to ignition of kerosene lamps used for lighting and/or kerosene stoves cooking. Since the worsening of the power crisis in Nigeria many families have turned to the use of the hurricane lantern and cooking stoves. In Ibadan, South Western Nigeria, Odeyinde[10] and colleagues also noted this upsurge in cases related to kerosene fuelled flames accounting for 43.5% of cases with petrol flame contributing another 4.8%. This is also similar to the report of Dongo, *et al*[11] in Irrua, Mid Western Nigeria, and Fatusi, *et al.*[12] in South Western Nigeria. However, earlier reports from North Western[4,13] and North Eastern[5] Nigeria suggests scalds to be the most common cause of paediatric burns; a pattern consistent with reports from the more developed economies. This may be related to cultural differences related to alternate power sources for lighting and cooking. We note that children below three suffer scald injuries more than flame burns, which may represent different levels of exposure. We did not come across any case of hot water bath injury. This is not commonly available in most homes.

Of those whose time of presentation relative to the incident injury could be evaluated, only eight presented within 24 hours. This certainly does not augur well for effective fluid resuscitation. It is possible that they may have sought aid from some other care givers prior to presentation. Thus we need to assess the knowledge and adequacy of fluid therapy post burns in among general practitioners in the city. Evidently also there is a need for public education not only on prevention but also on the need for prompt rescue and transfer of burn patients to specialized units. Dongo, *et al.*[11] found 60% of their burns cases presenting to the hospital within the first 6 hours supposedly because lower level care givers are unwilling to intervene in major burns. In the city, they appear more daring. In addition, we receive cases from both rural and urban centres, including the Irrua centre. It would appear that our centre is an option of last resort; consistent with the public perception of most teaching hospitals in Nigeria.

Burn extent in 82% of the patients assessed was 30% and below with many children sustaining between 11-20%TBSA burns. These are usually associated with good prognosis in this age group in the absence of significant co morbidities and inhalational injury. One challenge we face is the burden of infection control in the absence of the isolation facility provided by purpose built burn units. Though many of the injuries are predominantly partial thickness, wound sepsis converts them to full thickness cutaneous injury and increased need for skin grafts.

Burn wound sepsis was the most frequent complication seen in our series and it accounted for the two deaths recorded during the period. Late presentation to hospital, care in the open ward and perhaps breakdown in infection control protocols may account for this. It is hoped that with the controlled environment and skilled personnel in a burn unit, this can be reduced. Provision of early skin cover for deep burns will also help control septic complications as well as reduce hospital stay.

Much has been said for early wound excision and grafting in the management of deep burns[1]. However, the routine practice remains a challenge in most of the developing world. Lack of manpower, inadequate operating space and instrumentation, weak blood banking support, absent skin substitutes are among the numerous constraints. We have successfully managed available donor sites by using the tissue available to cover priority areas: joint areas, the face and hands where scar morbidities are more profound.

The best management of scars and contractures include prevention, appropriately timed surgery[14] and physiotherapy.

In conclusion, children constitute a vulnerable group for burns. Most injuries occur in the home setting where effective control measures can be adopted. Toddlers should be left all by themselves only in controlled play rooms where all health hazards have been screened for and excluded. Safety guidelines are essential for the use of kerosene and other flammables for lighting purposes. Safer lanterns and cooking stoves are already being designed. We may have to import them, copy the technology, or modify what we have to make them safer. A little more care and safety consciousness on the part of adults will also lead to some reduction in incidence. The availability of burn units and stimulation of interest in burn surgery will impact positively on outcome.
